# A Dual-Route Perspective of SARS-CoV-2 Infection: Lung- vs. Gut-specific Effects of ACE-2 Deficiency

**DOI:** 10.3389/fphar.2021.684610

**Published:** 2021-06-11

**Authors:** Elizabeth M. Sajdel-Sulkowska

**Affiliations:** Department of Psychiatry, Harvard Medical School, BWH, Boston, MA, United States

**Keywords:** COVID-19, ACE-2 receptor, gastrointestinal infection, microbiota, RAAS inhibitors

## Abstract

SARS-CoV-2, primarily considered a respiratory virus, is increasingly recognized as having gastrointestinal aspects based on its presence in the gastrointestinal (GI) tract and feces. SARS-CoV-2 uses as a receptor angiotensin-converting enzyme 2 (ACE-2), a critical member of the renin-angiotensin-aldosterone system (RAAS) involved in the regulation of blood pressure and fluid system. In addition to the systemic endocrine functions, RAAS components are also involved in intracrine and organ-specific local functions. The angiotensin-converting enzyme 2 (ACE-2) is a key component of RAAS and a receptor for SARS-CoV-2. It is expressed in many tissues with gastrointestinal (GI) tract ACE-2 levels far exceeding those in the respiratory tract. SARS-CoV-2 binding to its receptor results in a deficiency of ACE-2 activity in endocrine, intracrine, and local lung and GI tract ACE-2. The local ACE-2 has different organ-specific functions, including hypertension-independent activities; dysregulations of these functions may contribute to multiorgan COVID-19 pathology, its severity, long-term effects, and mortality. We review supporting evidence from this standpoint. Notably, COVID-19 comorbidities involving hypertension, obesity, heart disease, kidney disease, and diabetes are associated with gastrointestinal problems and display ACE-2 deficits. While RAAS inhibitors target both endocrine and intracrine ACE-2 activity, the deficit of the local ACE-2 activity in the lungs and more so in the gut have not been targeted. Consequently, the therapeutic approach to COVID-19 should be carefully reconsidered. Ongoing clinical trials testing oral probiotic bound ACE-2 delivery are promising.

## Introduction

COVID-19 pandemic has affected millions worldwide, with emerging new variants posing public health issues of unprecedented proportions. SARS-CoV-2 was initially recognized as a respiratory virus, spreading through the nasal passage to the upper and lower respiratory tract using, as a receptor, the angiotensin-converting enzyme 2 (ACE-2; Xiao et al., 2020), a critical member of the renin-angiotensin-aldosterone system (RAAS) involved in the regulation of blood pressure, fluid, and electrolyte balance, and vascular resistance. RAAS components, including ACE-2, are involved in systemic endocrine, intracrine and organ-specific local functions.

SARS-CoV-2 is increasingly recognized as a gastrointestinal virus based on its presence in the gastrointestinal (GI) tract and feces (Jin et al., 2020; Lin et al., 2020) Furthermore, the level of the ACE-2 receptor is remarkably high in the gastrointestinal (GI) tract. SARS-CoV-2 binds to its receptor, sequesters ACE-2 catalytic activity, and affects its vital physiological functions. ACE-2 deficit contributes to dysregulation of RAAS endocrine, intracrine and local activity both in the lung and in the gut. The situation is even more dramatic in individuals already burdened with several comorbidities involving hypertension such as pulmonary hypertension, diabetes, cardiovascular and kidney disease, which share dysregulated RAAS, including decreased ACE-2 activity that is further exacerbated by SARS-CoV-2 infection.

ACE-2 is a critical component of RAAS. It is also a key factor in the SARS-CoV-2 infection as the viral receptor in the lung and the gastrointestinal (GI) tract. ACE-2 has different organ-specific functions, including the hypertension-independent activity, associated with organ-specific expression of ACE-2-associated proteins and receptors (Cosarderelioglu et al., 2020). In the lungs, ACE-2 interacts with a specific bradykinin receptor B1 (BKB1R) regulating lung inflammation (Magrone et al., 2020). In the gut, ACE-2, in addition to BKB1R, interacts with the neutral amino acid transporter B^0^AT1 involved in the regulation of metabolism and local and systemic immunity (Broer, 2009). The overreaching question addressed in this review is whether the deficiency of the gut-specific ACE-2 functions is inflicting a more significant systemic effect? This review refers to the ACE-2 activity associated with the BKB1R or B^0^AT1 as local, organ-specific ACE-2 activity and considers relevant data from this standpoint.

The premise examined here implies that the local gut ACE-2 activity is critically involved in multiorgan COVID-19 pathology, its severity, long-term effects, and mortality. In contrast the local lung ACE-2 deficiency is critically related to lung inflammation and respiratory symptoms. The premise is examined in terms of gastrointestinal vs. respiratory symptoms, ACE-2 tissue distribution, and the impact of local ACE-2 deficiency in the gut vs. lungs.

Furthermore, we address the controversy regarding the effect of ACEIs and ARBs in the context of SARS-CoV-2 infection. We conclude with the review of the current therapeutics and explore potential new therapies. It is envisioned that the perspective presented here contributes to the understanding of the SARS-CoV-2 infection, management of RAAS dysregulations, and ACE-2 deficiency under different pathological conditions.

## COVID as a Gastrointestinal Infection

### Lung vs. gut Route of SARS-CoV-2 Infection

**FIGURE 1:**
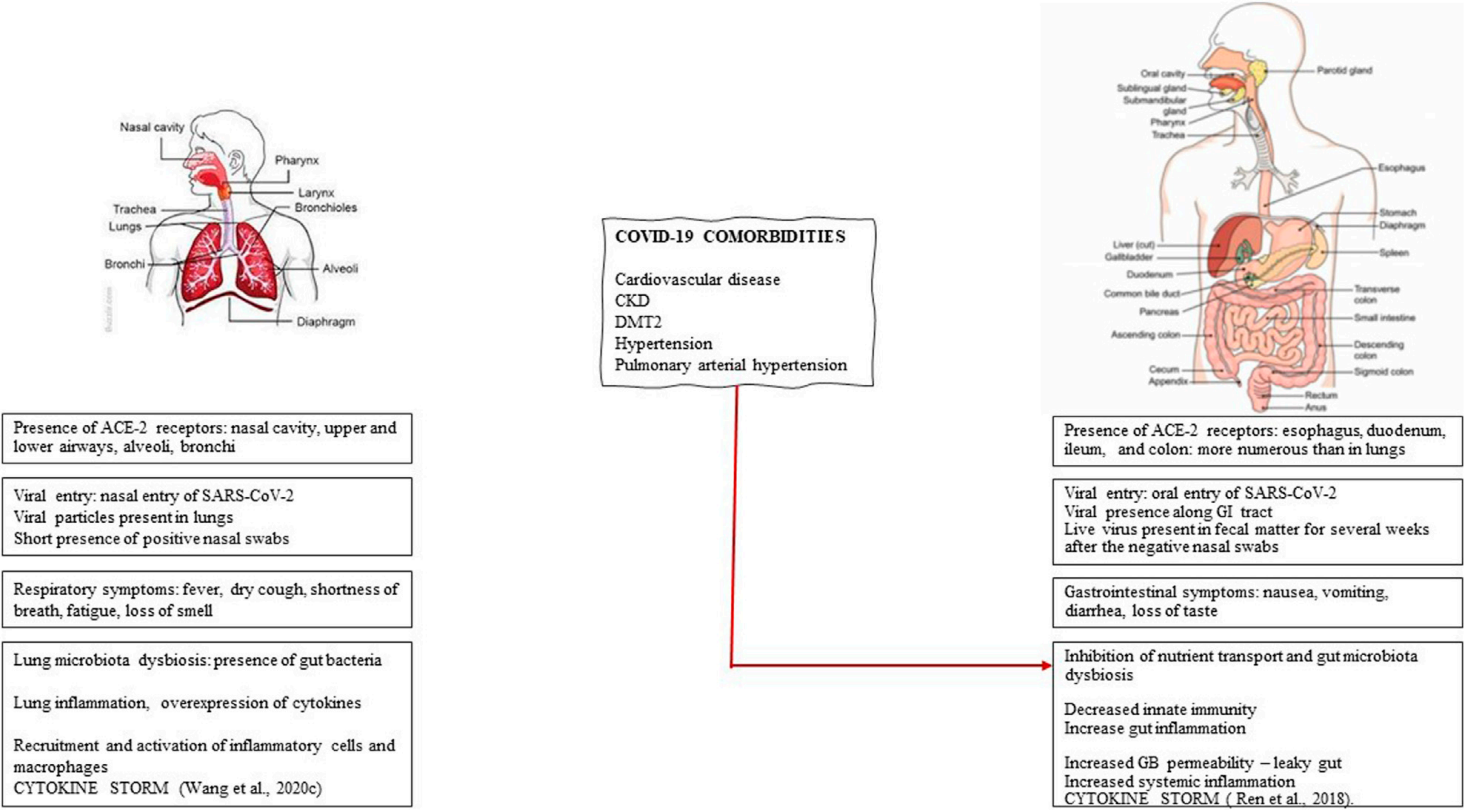
Respiratory vs. gastrointestinal aspects of Sars-CoV-2 infection. The respiratory and gastrointestinal nature of SARS-CoV-2 infection is compared in terms of the relative distribution of ACE-2 receptors, viral entry, presence of viral particles, symptoms, gut microbiota, and inflammation. The critical involvement of gut microbiota dysbiosis that determines the risk and the severity of COVID-19 is present in several comorbidities such as cardiovascular disease, chronic kidney disease (CKD), diabetes mellitus type 2 (DMT2), hypertension, and pulmonary arterial hypertension.

COVID-19, initially considered primarily a respiratory infection, has since been increasingly recognized for the associated gastrointestinal aspects; a comparison of respiratory vs. gastrointestinal features is presented in Figure 1. Accumulating evidence points to gastrointestinal symptoms such as constipation, abdominal pain, gastroesophageal reflux, and vomiting in 23–70% of patients (Jin et al., 2020; Lin et al., 2020). The GI symptoms are associated with gastroscopic data showing significant epithelial damage and numerous infiltrating plasma cells and lymphocytes in the esophagus, stomach, duodenum, and rectum (Lin et al., 2020; Xiao et al., 2020). Furthermore, comorbidities that increase both the risk and severity of COVID-19 are associated with gastrointestinal symptoms. Meta-analysis showed that the prevalence of severe COVID-19 is more common in patients with gastrointestinal symptoms (Cheung et al., 2020).

Further support for the gastrointestinal route of infection comes from the observations of SARS-CoV-2 viral particles presence in the GI and fecal excretion in half or more (Cheung et al., 2020) infected subjects for weeks even after negative nasopharyngeal results (Cardinale et al., 2020). Persistent detection of the virus in the fecal samples compared with the nasal cavity (Xiao et al., 2020) supports the perspective that the oral route of infection is more extensive than the nasal route. Importantly, the virus remains infectious in the sewage of hospitals for two weeks (Ding and Liang, 2020). From a public health perspective, the oral route of infection in general, and specifically fecal-mouth route, presents a risk of infections in places equipped with public toilets, such as in airplanes, ship cruises, and significantly preschool and public-school setups. Despite the high concentration of virus RNA in the stool, the evidence for the infectious virus has been limited (Wolfel et al., 2020). Rectal swabs revealed positive RT-PCR results, but no replication-competent virus was detected (Xu et al., 2020). However, live SARS-CoV-2 has been recently isolated from patients' stool in China (Gu et al., 2020; Xiao et al., 2020). Others detected live viruses in the feces of patients with COVID-19 and the feces of asymptomatic patients (Sun et al., 2020). Furthermore, a small percentage of blood samples also contained live viruses suggesting viral entry through the bloodstream and suggesting systemic infection with the virus (Wang et al., 2020a).

It has been suggested that SARS-CoV-2 gastrointestinal infection results in inflammation and diarrhea that affects up to 30% of cases (de Oliveira et al., 2020; Pan et al., 2020), and CT scans suggest that diarrhea may be linked to bowel wall abnormalities (Cardinale et al., 2020; Bhayana et al., 2020). Gut inflammation is supported by elevated fecal calprotectin (FC) in COVID19 patients with active or completed diarrhea (Effenberger et al., 2020). Interestingly SARS-CoV-2 RNA was not detected in the feces of patients with ongoing diarrhea but was detected in patients with completed diarrhea, suggesting that changes in gut permeability facilitate fecal RNA excretion. Changes in mucosal permeability and nutrient malabsorption could contribute to gut microbiota dysbiosis and diarrhea (de Oliveira et al., 2020). The loss of electrolytes and water could be amplified by dysregulated RAAS system. Changes in mucosal permeability of infected intestinal cells and malabsorption in the enterocytes could contribute to dysbiosis. However, autophagy regulated by intestinal microbiota may also be a contributing factor. It has been suggested that SARS-CoV-2 causes alterations in gut microbiota, which results in diarrhea (de Oliveira et al., 2020). The emerging scenario includes oral viral infection, gut microbiota dysbiosis, altered gut-blood barrier, systemic trafficking of gut metabolites, and the virus itself. In turn, the bidirectional gut-lung (GL) axis impacts the lung’s microbiota and lung immune response to the virus.

### COVID-19 Comorbidities

Both the risk and severity of COVID-19 are frequently associated with comorbidities such as systemic and pulmonary hypertension, cardiovascular diseases, chronic kidney diseases (CKD), diabetes mellitus type 2 (DMT2), obesity, pneumonia, and acute respiratory syndrome (ARDS). The risk and severity of COVID-19 also are gender (Liu N. et al., 2020) and age-dependent (Li et al., 2020). Importantly, these comorbidities are associated with microbiota dysbiosis, dysregulation of RAAS, and a deficit of ACE-2 activity. Accumulating evidence implicates gut microbiota in regulating ACE-2 expression and, as such, a contributing factor to the pathogenesis of cardiovascular diseases, intestinal inflammation, malnutrition, immunity, and energy metabolism. Thus, the higher risk and severity of infection in patients with comorbidities could be explained by the additive effect of ACE-2 deficiency. Furthermore, the effect on the local gut ACE-2 deficiency could be amplified in these patients.

Low levels of ACE-2 were reported in primary respiratory pathologies such as pulmonary hypertension, atherosclerosis (Tikellis and Thomas, 2012), and metabolic pathologies such as obesity (Iannelli et al., 2020) and DMT2, where the decreased ACE-2 activity was observed in the GI (Obukhov et al., 2020). On the other hand, others reported increased expression of ACE-2 levels in kidneys (Ye et al., 2004; Wysocki et al., 2006), serum, liver, and pancreas (Roca-Ho et al., 2017) in animal models of diabetes, and lung ACE-2 activity extrapolated from human data (Rao et al., 2020) supporting tissue-specific ACE-2 regulation (Riviere et al., 2005).

Several SARS-CoV-2 comorbidities are also associated with dysregulation of RAAS and hypertension. Therapies involving RAAS inhibitors, ACEIs, and ARBs, are directed at minimizing the effect of ACE-2 deficiency. As stated above, SARS-CoV-2 infection further decreases not only systemic but also local RAAS activity. In diabetes, the local gut RAAS-independent ACE-2 deficiency must be considered, as it is involved in the regulation of glucose, sodium, water, and amino-acid uptake by interacting with the neutral amino acid transporter (B^0^AT1). The decreased supply of nutrients affects gut microbiota resulting in gut dysbiosis and changes in gut microbiota leading to the diabetes-specific microbiota profile (Zabetakis et al., 2020). Gut dysbiosis, in turn, affects the gut associated lymphoid system (GALT); and overactivation of GALT may contribute to cytokine storm (Zabetakis et al., 2020).

Importantly, a decrease in ACE-2 activity in some COVID-19 comorbidities, such as hypertension and DMT2, may be related to a high degree of genetic polymorphism in the ACE-2 gene (Sayed, 2021). Specific ACE-2 single nucleotide polymorphism (SNP) variants have been related to the risk of T2DM (Wu et al., 2017), susceptibility to T2DM in Asian population (Burrell et al., 2004), risk of T2DM with hypertension, and risk of T2DM related to left ventricular remodeling (Liu et al., 2018). ACE-2 polymorphism should be considered while choosing RAAS inhibitors in COVID-19 with diabetes.

DMT2 increases the risk of poor outcomes in COVID-19, and 12–16% of individuals with a severe infection have diabetes (Obukhov et al., 2020). In diabetic individuals, SARS-CoV-2 may amplify vascular dysfunctions, cardiac and kidney diseases (Obukhov et al., 2020) due to increased vascular inflammation (Pacurari et al., 2014). Arteriosclerosis may also be increased in COVID-19 patients with diabetes or obesity due to dysregulation of the local RAAS system in vascular tissue, immune cells, and adipose tissue (Aroor et al., 2013).

Interestingly, gastrointestinal disorders have not been considered as comorbidities in COVID-19 despite observations of gut microbiota dysbiosis and decreased ACE-2 activity in the ileum of patients with Crohn's disease (Novak et al., 2020) and down-regulation of intestinal epithelial ACE-2 in IBD patients (Burgeno et al., 2020).

## ACE-2 Receptor as Part of Endocrine, Intracrine, and Local RAAS: Systemic vs. Local RAAS Activity in the Respiratory and the Gastrointestinal Tract

RAAS is a critical regulator of blood pressure, fluid and electrolyte balance, and vascular resistance. Composed of several regulatory components and effector-peptides, RAAS controls blood flow and trophic responses to various stimuli and regulates vascular functions in health and disease. In addition to the systemic endocrine functions, RAAS is expressed in different tissues and performs intracrine and tissue-specific functions. The endocrine and intracrine RAAS consists of two arms with opposing activities, vasoconstricting and vasodilating. Classical RAAS, comprised of ACE, Ang I, and angiotensin type I receptor (AT1), is involved in the conversion of angiotensin I (Ang I) by ACE1 to Ang II that is a ligand for both angiotensin receptors AT1 and AT2: when bound to the AT1 receptor, Ang II induces vasoconstriction resulting in hypertension, water retention, thirst response, cardio hypertrophy, tissue fibrosis, and inflammation. The alternative arm of RAAS, comprised of ACE-2, receptor AT2, and Mas receptor, is involved in converting Ang II bound to AT2 receptor to Ang 1–7 (Ang 1–7). Ang (1–7) upon binding to the Mas receptor promotes vasodilation, hypotension and has antithrombotic, antifibrotic cardioprotective, and anti-inflammatory effects. ACE-2 also plays an important role in regulating inflammation by opposing the action of Ang II in inflammatory processes. As a proinflammatory modulator, Ang II increases vascular permeability that initiates the inflammatory process, followed by recruitment of infiltrating cells into the tissues through direct activation of the inflammatory cells, macrophages or by regulation of the expression of adhesion molecules and chemokines by resident cells such as macrophages (Suzuki et al., 2000). ACE-2 also plays a significant role in immune regulation and immune response to COVID-19. Importantly, ACE-2 regulates both local and systemic inflammation.

RAAS exerts systemic endocrine effects throughout the body that involve ACE-2 bound to the vascular endothelial cells. Also, most organs, including kidneys, adrenal glands, liver, pancreas, reproductive organs, skin, heart, brain, lungs, and gut, express the components of RAAS, allowing for autonomous local action. The local RAAS exerts both intracrine and local tissue-specific effects.

As part of RAAS, ACE-2 exerts both systemic and organ-specific functions. In the lungs, ACE-2 participates in intracrine functions and interacts with a specific DABK/bradykinin receptor B1 (BKB1R) and plays a critical role in regulating lung inflammation. Gut's ACE-2, in addition to its intracrine-, and DABK/bradykinin axis-dependent activities, binds to B^0^AT1 and is involved in several metabolic functions, such as nutrient transport, regulation of protein metabolism, and digestion. It also interacts with gut microbiota and gut-associated lymphoid system (GALT), regulates inflammation, and modulates local and systemic immunity.

Importantly, ACE-2 bound to the cell membranes acts as a receptor for SARS-CoV-2. Cell entry of SARS-CoV-2 depends on the binding of viral spike (S) to ACE-2 and S protein priming by cell membrane bound transmembrane serine protease 2 (TMPRSS2) expressed by endothelial cells across the respiratory and GI tracts (Hoffman et al., 2020). Recently, another membrane-bound protease, furin, has been shown to activate SARS-CoV-2 by cleaving the S protein at the specific S1/S2 cleavage furin-like cleavage site (FCS) not present in SARS-CoV, increasing viral infectivity (Jaimes et al., 2020). The fusogenic capacity of SARS-CoV-2S can be increased by trypsin present in many tissues, including GI epithelial cells (Soreide et al., 2006; Rolland-Fourcade et al., 2017). However, human airway trypsin-like protease (HAT) is less efficient (Xia et al., 2020). It is thus possible that SARS-CoV-2 binding, and cell entry are more efficient in the gut.

Upon binding and entering the cell, SARS-CoV-2 sequesters ACE-2 and leads to ACE-2 deficiency that results in systemic and organ-specific effects. Human ACE-2 shows significant homology with bat ACE-2, the most likely source of infection, but low homology with mice or rats, limiting the scope of animal models.

## ACE-2 Distribution; Local ACE-2 Activity in the Respiratory and Gastrointestinal Tract

ACE-2 receptors are ubiquitously expressed in the organism. They are expressed in the epithelial cells in the lungs, enterocytes of the small intestine and colon, proximal tubular cells of the kidney, neuronal and glial cells in the brain, vascular endothelial cells, smooth muscle cells, and cardiomyocytes (Garg et al., 2012). The expression of ACE-2 receptors is most abundant in the intestine and kidney, followed by testis, the heart and lungs, and is more abundant in the GI tract than in the respiratory tract (Wang et al., 2020b).

In the respiratory tract, ACE-2 receptors are localized in the nasal cavity and upper and lower airways. They are highly expressed in the apical membranes of cells in the sinonasal cavity (Gengler et al., 2020). In the lungs, ACE-2 receptors are present in type I and type II alveolar epithelial cells, bronchiolar epithelial cells, endothelial cells, and arterial smooth muscle cells (Hamming et al., 2007).

In the gastrointestinal tract, ACE-2 receptors are highly expressed in the esophageal upper and stratified epithelium, in the brush-border on the apical surface of mature enterocytes of the duodenum, and ileum (Garg et al., 2012; Ding and Liang 2020; Zhang H. et al., 2020), and are highly expressed in mesenteric microvascular endothelium of the colon (Zhang H. et al., 2020). ACE-2 receptors are important regulators of intestinal inflammation and are more numerous in the ileum, duodenum, and colon than in the lung. Importantly, ACE-2 receptors increase with age in the duodenum (Zhang H. et al., 2020). Colonic ACE-2 receptors are regulated by microbiota (Yang et al., 2015).

In addition to the membrane-bound vascular epithelium, a soluble form of ACE-2 (sACE-2) is present in plasma and urine. The sACE-2 can bind the virus, but the complex cannot enter the cell due to a lack of interaction with TMPRSS2 essential for entering SARS-CoV-2 into cells (Hoffman et al., 2020). Interestingly sACE-2 retains catalytic activity and can convert circulating Ang II to Ang-(1–7) and increase the systemic protective effects (Leow, 2020). The sACE-2 is derived by shedding from the membranes due to the hydrolyzing activity of a membrane-anchored metalloproteinase 17 (ADAM17; Patel et al., 2014). ADAM17, involved in proinflammatory processes in epithelial and endothelial cells, is increased in several pathologies, including cardiopulmonary, and renal systems and gastrointestinal inflammatory diseases such as ulcerative colitis (Rahman et al., 2021). ACE-2 deficiency due to SARS-CoV-2 binding to the ACE-2 receptor, activates ADAM-17 (Andring et al., 2020).

### ACE-2 Activity in the Lungs

Intracrine lung ACE-2 activity lowers Ang II, plays a critical role in regulating inflammation (Kuba et al., 2006), and protects against acute lung injury in several animal models of ARDS. Also, local lung ACE-2 is crucial in the pathogenesis of acute lung inflammation partly through modulating BKB1R axis signaling (Magrone et al., 2020). Evidence derived from animal studies points out to DABK as a biological substrate of ACE-2 in the lungs; loss of ACE-2 function may activate the BKB1R axis and contribute to lung inflammation and injury.

### ACE-2 Activity in the GUT

Intracrine gut ACE-2 activity involves the regulation of gastrointestinal epithelial fluid, electrolyte homeostasis, smooth muscle control, GI mucosal inflammation, and gut-specific fibrosis (Garg et al., 2012). Local gut ACE-2 functions include activity associated with the BKB1R axis. In addition, it includes B^0^AT1-dependent activity, which is not expressed in the lungs (Broer, 2009). In the gut, B^0^AT1 shares a location with ACE-2 in small intestine brush border apical membranes and acts as a chaperone for membrane trafficking of B^0^AT1 and mediates the uptake of glutamine and tryptophan into intestinal cells in a sodium-dependent manner. It also regulates glucose, fluid and electrolyte absorption and secretion, motility, and inflammation. In turn, tryptophan is required for optimal immune response, such as T-cell proliferation (Ren et al., 2018). It also promotes anti-inflammatory status, preserves tight junctions, decreases mucosal cell autophagy, and increases antimicrobial peptides through the mTOR pathway. Antimicrobial peptides influence the composition of gut microbiota. ACE-2 receptor also regulates the expression of B^0^AT1 in the intestine (Camargo et al., 2020).

## Effect of SARS-COV-2 Associated ACE-2 Deficiency in Lungs vs. GUT

**FIGURE 2:**
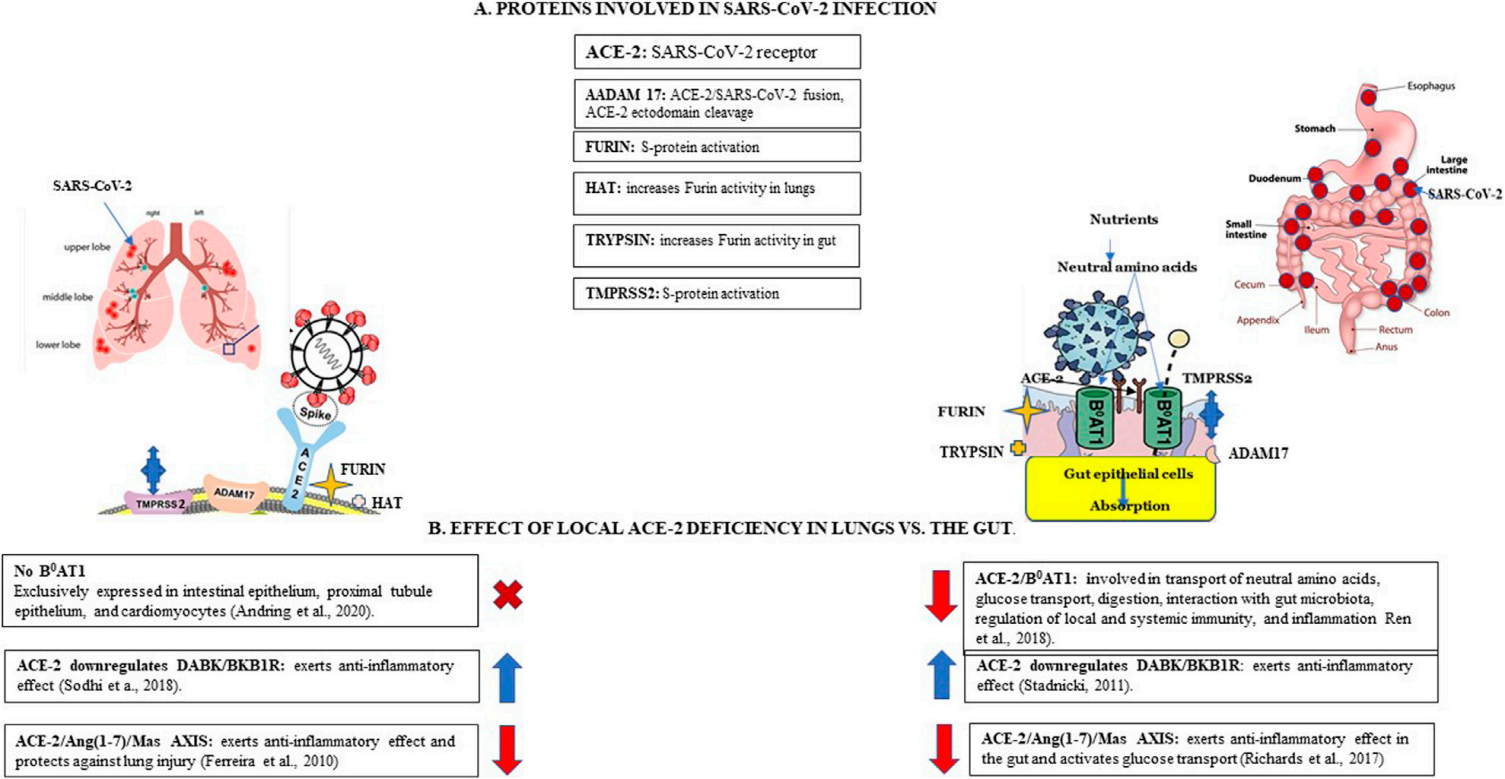
SARS-CoV-2 infection and local ACE-2 deficiency in lungs vs. the gut. Panel **(A)**. Proteins involved in SARS-CoV-2 infection*.* Angiotensin cleaving enzyme 2 (ACE-2) functions as SARS-CoV-2 receptor, expressed more abundantly in the gut than lungs. A membrane-anchored metalloproteinase 17 (ADAM17) is involved in shedding sACE-2 from membranes, may be involved in activation of SARS-CoV-2 spike (S) protein. Furin, a membrane-bound protease activates SARS-CoV-2 by cleaving S-protein at the specific furin-like cleavage site (FCS). Trypsin increases SARS-CoV2 fusogenic capacity in the gut. Human airway trypsin-like protease (HAT) increases viral fusogenic activity in the lungs but is less efficient than trypsin. Membrane-bound transmembrane serine protease 2 (TMPRSS2) is involved in S-protein priming. Panel **(B)**. Effect of local ACE-2 deficiency in lungs vs. the gut*.* ACE-2, functions as a chaperone for the neutral amino-acid transporter B^0^AT1 in the gut; B^0^AT1 is not expressed in the lungs. ACE-2 deficiency in the gut results in down-regulation of ACE-2/B^0^AT1 complexes and decreased intestinal uptake of neutral amino acids such as glutamine and tryptophan, critical to T-cell functions thus contributing to inflammation; decreased tryptophan transport interferes with glucose homeostasis. ACE-2 interacts with DABK/bradykinin receptor B1 (BKB1R) and plays a critical role in regulating inflammation *via* ACE-2/DABK/BKB1R axis; ACE-2 deficiency contributes to increased inflammation in lungs and the gut. ACE-2 interacts with the MAS oncogene (Mas) receptor which activates glucose transport *via* ACE-2/Ang-(1–7)/Mas axis; ACE-2 deficiency blocks glucose transport in the gut (Ferreira et al., 2010; Stadnicki, 2011; Richard et al., 2017).

SARS-CoV-2, following binding to its receptor and activation by the TMPRSS2, sequesters ACE-2 activity and affects its vital physiological functions by shifting from the protective arm of RAAS to the deleterious RAAS arm (Penninger et al., 2021). ACE-2 deficiency contributes to dysregulation of RAAS endocrine, intracrine, and local activity both in the lung and in the gut. Increased ANG II levels contribute to hypertension, increased inflammation, and vascular permeability, allowing virus spreading. As already mentioned, TMPRSS2 is essential for the entry of SARS-CoV-2 into cells by activating spike proteins; this activation requires ACE-2 receptor bound to the cell membrane. TMPRSS2 is expressed both in the lungs and in enterocytes in the esophagus and small intestine, where it is co-expressed with ACE-2 (Lee I. V. et al., 2020). A summary of proteins participating in SARS-CoV-2 infection and local ACE-2 deficiency in lungs vs. gut is presented in Figure 2.

### ACE-2 Deficiency in the Lungs

A reduction in pulmonary ACE-2 activity contributes to vascular lung inflammation, acute lung injury, and may contribute to cytokine storm (Wang et al., 2020c) because of the overexpression of cytokines and recruitment and activation of inflammatory cells such as macrophages. Patients with COVID-19 have shown interstitial mononuclear inflammatory infiltrates dominated by lymphocytes in the lungs followed by cytokine-storm-driven inflammation (Wang et al., 2020b; Wang et al., 2020c). As mentioned before, DABK/BKB1R is a biological substrate for ACE-2 (Sodhi et al., 2018). Thus, a decrease in ACE-2 results in impaired ability to inhibit BKB1R axis-mediated signaling, resulting in the more rapid onset of neutrophil infiltration and more severe inflammation in the lung.

### ACE-2 Deficiency in the GUT

SARS-CoV-2 infection downregulates the level of luminal ACE-2 that impacts several processes, such as nutrient transport, gut permeability, and local and systemic inflammation. It results in increased Ang II and decreased Ang1-7 levels in the luminal surface of enterocytes. In turn lower Ang (1–7) results in activation of AT1R, enhanced gut permeability associated with the leaky gut syndrome (Perlot and Penninger, 2013), and increased systemic inflammation. Leaky gut may also facilitate cytokine storms (Ren et al., 2018). Evidence suggests that the deficiency of ACE-2 and increased activity of Ang II-AT1 complexes are involved in the pathogenesis of inflammatory bowel disease (IBD; Fandriks, 2011).

ACE-2 deficiency results in down-regulation of ACE-2/B^0^AT1 complexes and decreased intestinal uptake of neutral amino acids such as glutamine and tryptophan. These amino acids are critical to T-cell functions and are involved in activating the innate immune system, such as TLR signaling and activation of NF-kB, and contribute to inflammation (Ren et al., 2018). Tryptophan, a serotonin precursor, also activates incretins that enter the circulation to modulate glucose homeostasis and contribute to hypoglycemia. Reduced binding of ACE-2 to Mas receptors blocks glucose transport in the gut by modulating luminal glucose transporters, SGLT1 and GLUT. ACE-2 deficiency in the luminal surface of enterocytes also participates in the degradation of digestive enzymes to yield free amino acids and influence bacterial metabolism.

Data derived from knockout ACE-2 mice suggest that the absence of ACE-2 decreases activation of the mTOR pathway reducing the secretion of antimicrobial peptides, alters gut microbiota, and increases susceptibility to inflammation that can be reversed by the administration of ARB irbesartan (Lu et al., 2018). Impaired amino acid transport and reduced secretion of antimicrobial peptides by Paneth cells in the small intestine affect innate immunity and contribute to colitis (Camargo et al., 2020) or IBD-like symptoms (Camargo et al., 2020; Nisoli et al., 2020). Accumulation of neutral amino acids in the intestinal lumen brings about microbiota changes, immune dysregulation and promotes diarrhea. These changes occur in rear genetic Hartnap disease with a mutation in B^0^AT1 characterized by protein malabsorption, microbiota changes, and immune defects (Camargo et al., 2020). Furthermore, lower levels of antimicrobial peptides lead to increased pathogen levels, altered gut microbiota, and dysbiosis, contributing to diarrhea in COVID-19 (Yang et al., 2015).

It has been previously suggested that ACE-2 deficiency in COVID-19 contributes to increased inflammation and cytokine storms through increased Ang II levels, decreased ACE-2/MasR axis activity, and activation of the BKB1R axis and the complement system (Kim et al., 2020). However, the effect of ACE-2 deficiency on the B^0^AT1-dependent activity has not been sufficiently examined. Importantly, B^0^AT1 is expressed only in the GI and kidney, but not in lungs. Furthermore, the sheer volume of luminal surface of the gut and the involvement of B^0^AT1 make the gastrointestinal aspects of SARS-CoV-2 infection more consequential.

## Local gut ACE-2 ACTIVITY and gut Microbiota

The gut microbiota plays a significant role in human health and disease (Sajdel-Sulkowska, 2019). Changes in the composition of gut microbiota contribute to hypertension (Tanaka and Itoh, 2019) and are common in several COVID-19 comorbidities, such as DMT2 (Allin et al., 2015), kidney disease (Chen et al., 2019), hypertension, and cardiovascular diseases (Segal et al., 2020). Evidence is accumulating that implicates the gut microbiota dysbiosis in the pathogenesis, severity, and disease course of COVID-19. The severity of COVID-19 is increased in the older population, which may be related to reduced bacterial diversity and enrichment in pro-inflammatory commensals with age (Ragonnaud and Biragyn, 2021).

Furthermore, gut microbiota regulates the expression of ACE-2 and thus determines the severity and infectivity of COVID-19. Gut microbiota may be a relevant target for the treatment of COVID-19, such as antiviral therapy or increased vaccine efficacy. Microbiota interaction with ACE-2 receptors is bidirectional. ACE-2 impacts the gut microbiota by regulating the transport of neutral amino acid and glucose and controlling antimicrobial peptides' secretion and intestinal inflammation Zhang H. et al., 2020). Microbiota, in turn, regulates colonic ACE-2 receptors (Yang et al., 2015; Segal et al., 2020; Wang et al., 2020c). Interestingly, deficiency of ACE-2 in the mouse gut was associated with a reduction in fecal commensal bacteria (Geva-Zatorsky, 2017). Evidence suggests a potential role of gut microbiota in susceptibility, progression, and severity of COVID-19 (Yeoh et al., 2021).

Gut microbiota is continuously replenished by the ingestion of external microbes and is regulated by the diet. This continuous exposure and exchange between gut microbiota and the external environment help maintain the gut symbiosis and diversity associated with healthy status. Under symbiotic conditions, most of the opportunistic bacteria are outcompeted by commensal organisms.

SARS-CoV-2 infection results in gut microbiota dysbiosis due to dysregulation of intestinal nutrient transport. Gut-microbiota dysbiosis increases gut-blood barrier permeability, activates GALT, and increases inflammation. Gut microbiota dysbiosis is likely responsible for cytokine storm leading to multiorgan failure. Furthermore, gut microbiota impacts lung microbiota in COVID-19 with a change in composition involving an increased proportion of gut species.

Indeed, GI symptoms observed in COVID-19 are closely related to abnormal gut microbiota. Several groups have used human fecal samples to examine changes in gut microbiota in COVID-19. Some studies showed a decrease in biodiversity and beneficial bacterial species (Dhar and Mohanty, 2020). Others show changes in gut microbiota in COVID-19 characterized by a depletion of health-promoting bacteria and enrichment of opportunistic proinflammatory pathogens, frequently associated with inflammatory bowel syndrome (IBD, He et al., 2020).

A significant reduction of bacterial diversity and an increase in opportunistic pathogens was observed in hospitalized patients in China (Gu et al., 2020) and COVID-19 associated increase in several bacterial species was concordant with disease severity (Yeoh et al., 2021; Zuo et al., 2020). Some bacterial species could distinguish between COVID-19 and healthy controls (Gu et al., 2020; Segal et al., 2020). Several bacterial species were associated with fecal viral load. Importantly, changes in gut microbiota persist after clearance of SARS-CoV-2 and resolution of respiratory symptoms (Zuo et al., 2020).

Interestingly, hallmark symptoms of SARS-CoV-2 infections include anosmia (complete loss of smell) and dysgeusia (lack of taste), linked not only to the disturbance in nasal microbiota (Koskinen et al., 2018) but also extra-nasal receptors regulated by gut microbiota composition (Bienenstock et al., 2017). Importantly they have been shown to control emotions such as fear that individuals with COVID-19 experience. Dysgeusia is related to abnormal activity of taste receptors (T2Rs) in the oral cavity and in the colon, where they are involved in the regulation of GI functions, such as GI motility, appetite, nutrient uptake, and fluid secretion. T2Rs, both in the mouth and the colon, are regulated by SCFAs produced by gut bacterial fermentation (Turner et al., 2018).

## RAAS Inhibitors: ACEIS and ARBS do not Address the Local gut ACE-2 Deficiency

RAAS inhibitors, ACEIs, and ARBs are used in pathologies involving dysregulated RAAS activity, such as hypertension and DMT2. ACEIs such as benazepril and captopril inhibit ACE activity and block the conversion of Ang I to Ang II, resulting in lower Ang II levels. ARBs, such as losartan or valsartan, prevent Ang II vasoconstricting activity by blocking the binding of Ang II to the AT1 receptor. Part of the benefit of ACEIs and ARBs treatment may be due to the diversion of RAAS activity toward Ang (1–7) pathway with subsequent Mas receptor activation (Garg et al., 2012) and vasodilatory, ant-inflammatory effects.

The potential therapeutic use of RAAS inhibitors in COVID-19 has been under heated debate brought about by observations suggesting that they may contribute to the increase in ACE-2 receptors and potentially increase the level of viral binding sites. Evidence for RAAS inhibitor-dependent upregulation of ACE-2 appears to be limited to animal studies, primarily rodents. Intravenous infusion of ACEIs or ARBs showed decreased plasma Ang II and blood pressure and increased ACE-2 mRNA in the circulation (Ishiyama et al., 2004; Ferrario et al., 2005; Diaz, 2020) while ARB increased both the mRNA and activity of ACE-2 (Ferrario et al., 2005). ACEI, captopril-treated mice showed reduced ACE-2 activity and protein levels in kidney and lung lysates; the decrease in kidney membrane-bound ACE-2 was accompanied by a significant systolic increase ACE-2 protein (Wysocki et al., 2020). In the ARB telmisartan-treated mice, there was a decrease in membrane-bound ACE-2 (Wysocki et al., 2020). Neither one had a significant effect on the lungs’ lysate or membranes (Wysocki et al., 2020). A review of 11 rat studies showed an increased level of ACE-2 on ACEI or ARB in kidney, heart, and plasma in half of the studies. SARS-infected animals showed that Losatran lowered blood pressure but significantly elevated ACE-2 in the heart and kidney (Klimas et al., 2015). ARB telmisartan resulted in increased ACE-2 in the renal vasculature (Soler et al., 2009).

In summary, the results of animal studies are not very consistent, and importantly not relevant to humans. Because of the relatively low sequence homology between rat and mice and human ACE-2, rodents are difficult to infect with the SARS-CoV-2 virus. Except for non-human primates, the only good small animal model of COVID-19 is a golden hamster, where binding of the virus to ACE-2 in the lungs results in infection (Imai et al., 2020), while the gastrointestinal viral effect has not been carefully addressed. Consequently, any data obtained from rodent studies on RAAS inhibitors should not serve as a base for human therapeutic use.

Analysis of human intestinal biopsies taken during routine gastroduodenoscopy and ileocolonoscopy showed that only ACEIs increased intestinal levels of ACE-2 and B^0^AT1, but no effect was seen with ARBs (Vuille-dit-Bille et al., 2014). In another study, ACEI increased cardiac ACE-2 mRNA but did not affect ACE-2 activity (Mourad and Levy, 2020).

Much confusion regarding the upregulation of ACE-2 in COVID-19 comes from the presence of sACE-2 in circulation, presumably due to a compensatory response or dimerization of ACE-2, which lacks affinity for SARS-CoV-2 binding (Yan et al., 2020). Importantly the soluble sACE-2 can bind the virus, but the complex cannot enter the cell; thus, an increase in the soluble form of ACE-2 does not seem likely to contribute to the infection's severity. However, this soluble sACE-2 could act as a decoy to bind the virus and decrease the likelihood of viral binding to membrane-bound ACE-2. It has been recently suggested that the sACE-2 retains catalytic activity and that the sACE-2-virus complex could interact with TMPRSS2 and thus contribute to infection (Rahman et al., 2021). Increased sACE2 activity was observed in patients with heart failure (Epelman et al., 2008). Some human studies with ARBs suggest upregulation of sACE-2 in hypertension (Furuhashi et al., 2015). It has been speculated that once the viral endocytosis occurs, there is a reduction in ACE-2 activity that results in Ang II accumulation and increased inflammation. This sequel could be addressed by treatment with RAAS inhibitors.

Furthermore, a hypothesis that unoccupied AT2 receptors may contribute to increased viral infectivity has not been supported by clinical observation. In data derived from the eleven clinical studies on ACE-2 protein or activity in serum or urine, seven showed no association between ACE-2 expression and the use of ACEIs or ARB (Sriram and Insel, 2020). Several clinical studies that reported an increase in ACE-2 did not specify which RAAS inhibitor was used (Liu Y. et al., 2020). Several studies reported no effect of RAAS inhibitors on ACE-2 levels in patients with COVID-19 (Henry et al., 2020).

Importantly, a special report on RAAS inhibitors (Vaduganathan et al., 2020) concluded that there is presently insufficient data to determine whether RAAS inhibitors increase ACE-2 expression in humans. The report recommended that withdrawal of RAAS inhibitors in high-risk groups may have adverse health effects and should be continued in patients who are in COVID 19 risks groups. A landmark Chinese study of patients with COVID-19 with hypertension showed that the use of ACEIs/ARBs was associated with a lower risk of mortality and lower risk of digestive system involvement, although it was not clear which particular RAAS inhibitors were used (Tan et al., 2020), while another Chinese study showed no effect (Li et al., 2020). Other studies showed that ARBs use was associated with reduced risk of severe COVID-19, morbidity, and mortality (Liu Y. et al., 2020). Others observed a tenfold decrease in mortality on ACEIs/ARBs (Zhang P. et al., 2020) and reduced inflammation in severe cases (Meng et al., 2020). On the other hand, a comparison of ACEIs and ARBs in hospitalized COVID19 patients showed that ACEIs exposure significantly reduced the risks of severe disease while ARBs had no effect (Bean et al., 2020; Senkal et al., 2020). An ARB, losartan was more effective in blocking the actions of Ang II than ACEIs and was also effective in controlling BP; however, it appears to affect smooth muscle contractility and may contribute to adverse effects (Patten and Abeywardena, 2017).

A review of international clinical data regarding ACEIs, and ARBs use in COVID-19 consistently demonstrated that they do not increase the risk of infection. Furthermore, the use of ACEIs and ARBs in antihypertensive therapy reduced the severity of COVID-19 and lowered the risk of mortality. Most importantly, there is no clinical evidence for ACEIs or ARBs predisposing patients to SARS-CoV-2 infection. Thus, human studies support the recommendations of the European Society of Cardiology, American Heart Association, American College of Cardiology, and the Heart Failure Society of America that patients with cardiovascular therapies should continue therapies with ACEIs/ARBs as clinically indicated (Chung et al., 2020).

However, as most of the studies focused strictly on the respiratory aspects of SARS-CoV-2 infection, more research is needed to understand the effect of RAAS inhibitors on the gastrointestinal aspects of COVID-19.

## Future Therapeutics: Embracing the Local ACE-2 Activity

Recombinant human ACE-2 (rhACE-2) has been considered a potential treatment for COVID-19 and examined for its ability to bind to the SARS-CoV-2. Several clinical trials have shown the safety of infusion in healthy candidates and well as patients with or without comorbidities. Other clinical trials are testing the ability of rhACE-2 to block viral entry and decrease viral replication (University NCT04287686 and ClinicalTrials.gov Identifier: NCT04335136). Treatment with rhACE-2 has been found to attenuate pulmonary hypertension (Treml et al., 2010). Furthermore, rhACE-2 activates the Mas receptor, resulting in cardiac output improvement, reduced inflammation, and protection from pulmonary injury. However, so far, there is no evidence of its activity in the GI tract (Batlle et al., 2020). The observation that sACE-2 can convert circulating Ang II to Ang-(1–7) and increase the systemic protective effects has been expanded to recombinant human rhACE-2 to curb coronavirus invasion (Pang et al., 2020).

An intriguing new treatment involves encapsulated ACE-2 and ACE-2/Ang (1–7) in complex with probiotics. Orally deliverable complexes increase ACE-2 and Ang (1–7) in the blood and offer protection against pulmonary hypertension (Shenoy et al., 2014). The treatment increased pulmonary ACE-2 levels and AT1 receptors and resulted in a reduced level of proinflammatory cytokine. It also upregulates B^0^AT1 and upholds communication between gut epithelia and microbiota (Sharma et al., 2020a).

A recent version of this approach used commensal *Lactobacillus paracasei* (LP) as a live vector for oral delivery of human ACE-2. LP expressing ACE-2 fused to a nontoxic subunit B of cholera toxin allowed transmucosal transport resulting in increased ACE-2 activity in serum and tissue. It has been shown to reduce inflammation in diabetic retinopathy in mice (Verma et al., 2019), upregulate B^0^AT1, and uphold epithelial–gut communication (Sharma et al., 2020b).

Interestingly, orally administered ACE-2/probiotic complexes travel *via* the GI tract and are absorbed in the gut. The question is whether it can interact with B^0^AT1 and increase the transport of neutral amino acids? If so, future therapeutics may use different probiotics with anti-inflammatory properties to reverse ACE-2 deficiency not only in COVID-19 but also in other diseases characterized by ACE-2 deficiency. Furthermore, the use of commercially available probiotics supporting gut and immune activity opens a gate for future use of other probiotics to restore gut microbiota, GALT activity and regulate systemic inflammation.

## Conclusion

The evidence discussed here points out to dual aspects of SARS-CoV-2 infection affecting both respiratory and gastrointestinal systems. It further supports the premise that while the deficiency of local lung ACE-2 activity is critically related to the regulation of lung inflammation, it is the local gut ACE-2 that is involved in multiorgan COVID-19 pathology, its severity, long-term effects, and mortality. Furthermore, a discussion is focused on the distinction between endocrine, intracrine and local organ-specific RAAS and lung-vs. gut-specific effects of ACE2 deficiency. The deficiency of ACE-2 in COVID-19 has been previously linked with increased inflammation and cytokine storm due to increased Ang II levels, decreased production of Ang (1–7), through Ace-2/Ang (1–7/MasR axis, and increased activation of BKB1R axis. However, the effect of ACE-2 deficiency on the gut-specific B^0^AT1-dependent activity, in the context of SARS-CoV-2 infection has not been sufficiently emphasized. The sheer volume of the luminal surface of the gut, the high level of ACE-2 receptors, and the involvement of B^0^AT1 make the gastrointestinal aspects of SARS-CoV-2 infection more consequential. Future therapies combining rhACE-2 with probiotics should be the focus in combating COVID-19.
